# Quantitative Assessment of Postural Influence on Lung Function Using Deformable Image Registration-Based Breath-Hold CT Ventilation Imaging

**DOI:** 10.7759/cureus.75900

**Published:** 2024-12-17

**Authors:** Morikazu Amano, Yoshihiro Kawai, Takaaki Ito, Hajime Monzen, Tsuyoshi Okawa, Eriko Sato

**Affiliations:** 1 Department of Radiation Therapy, Fujieda Municipal General Hospital, Fujieda, JPN; 2 Department of Radiological Technology, Fujieda Municipal General Hospital, Fujieda, JPN; 3 Department of Medical Physics, Graduate School of Medical Sciences, Kindai University, Osakasayama, JPN

**Keywords:** breath hold ct ventilation imaging (bh-ctvi), deformable image registration (dir), deformable vector field (dvf), four body positions, lung pressure

## Abstract

Purpose

This study aimed to clarify which positions are beneficial for patients with pathological lung diseases, such as acute respiratory distress syndrome, by obtaining lung ventilation and deformable vector field (DVF) images using Deformable Image Registration (DIR).

Methods

Thirteen healthy volunteers (5 female, 8 male) provided informed consent to participate to observe changes in normal lungs. DIR imaging was processed using the B-spline algorithm to obtain BH-CTVI (inhale, exhale) in four body positions (supine, prone, right lateral, left lateral) using DIR-based breath-hold CT ventilation imaging (BH-CTVI). DVF imaging was created through DIR-based BH-CTVI, which obtained the displacement vector from expiration to inspiration for each lung lobe.

Results

In the DIR images for each body position, the areas with Jacobian values in the 75th percentile or higher, indicating highly functional areas, were distributed on the side of the patient in contact with the ground. DVF images showed the abdominal displacement vector to be oriented from dorsal to ventral in the supine position. However, in the prone position, the displacement vectors were nearly parallel to the ground, directed from head to feet, indicating that lung motion was unaffected by gravity.

Conclusion

We demonstrated that the prone position allows for lung ventilation with the least gravitational load compared with the supine, right lateral decubitus, and left lateral decubitus positions, based on a comparison of DIR-based BH-CTVI when the positions were converted. It is important to include the evaluation of DVF images, in addition to ventilation images, when assessing lung function using DIR-based BH-CTVI.

## Introduction

Since the 1970s, turning patients with severe acute respiratory distress syndrome (ARDS) from a supine to a prone position has been a common practice. This intervention has been shown to improve lung function and reduce mortality rates [1,2]. Additionally, placing patients with ARDS in a prone position has been reported to improve gas exchange, alveolar ventilation, blood flow distribution, and the ventilation/perfusion ratio [3-5]. Various nuclear medicine imaging techniques, such as magnetic resonance imaging (MRI) using hyperpolarized noble gases and xenon-enhanced computed tomography (CT), are used to understand the pathophysiology of lung diseases. However, these methods are limited by low resolution, high costs, and lengthy scan times [6-8]. Although high spatial resolution four-dimensional (4D) CT-based techniques are frequently used, they present challenges such as artifacts from respiratory phase misalignment and increased radiation exposure [9-11].

Deformable Image Registration (DIR)-based breath-hold CT ventilation imaging (BH-CTVI) uses expiration and inspiration phase images to detect signal intensity changes within the lungs. This is achieved by comparing the expiration phase CT image to the inspiration phase CT image, which is deformed toward the expiration phase image [12-15]. DIR-based BH-CTVI can produce high-resolution lung ventilation images without the need for contrast agents, at a lower cost, with shorter scan times, and less radiation exposure than 4D-CT-based techniques [12-15]. Previous studies have shown that DIR-based BH-CTVI correlates well with lung function and can be applied to radiotherapy planning as a method for lung ventilation imaging [12]. Additionally, Shin et al. demonstrated that a comparison of deformable vector field (DVF) imaging in supine and prone positions, using expiration and inspiration images, can provide insights into the advantages of prone positioning for patients with pathological lung conditions such as ARDS [14]. Understanding the changes in lung ventilation and DVF imaging across different positions is clinically important, as positional changes, such as moving to the lateral decubitus position, may alter the direction and magnitude of the lung lobe displacement vectors.

This study aimed to assess lung ventilation and DVF images using DIR-based BH-CTVI in supine, prone, right lateral, and left lateral positions to elucidate why the prone position is beneficial for patients with pathological lung diseases, such as ARDS.

## Materials and methods

Data sets and image acquisition

Thirteen healthy volunteers (5 female, 8 male) provided informed consent to participate in this study from August 1, 2021, to September 30, 2021. Their characteristics are presented in Table 1. The study was conducted in accordance with the Declaration of Helsinki and was approved by the Medical Ethics Committee of our hospital facility (R03-6).

**Table 1:** Participant characteristics.

Variable	Mean ± SD
Age (years)	45.3 ± 7.9
Height (cm)	167.5 ± 10.3
Weight (kg)	62.5 ± 14.3
Body mass index (kg/m^2^)	21.8 ± 4.0

All participants underwent 320-slice multidetector CT scans (Aquilion One, Canon Medical Systems, Otawara, Japan) during natural inspiration and expiration. The CT parameters were as follows: tube voltage of 120 kVp, tube current of 100 mAs, slice thickness of 2 mm, reconstruction interval of 2 mm, FOV of 36 cm, matrix size of 512 × 512, and rotation time of 0.5 s.

Acquisition of lung ventilation and DVF images through positional transformation

The treatment planning system, Monaco (Elekta, Stockholm, Sweden), was used to automatically contour the lung fields on the expiratory and inspiratory CT images. DIR-based BH-CTVI deformed the inspiratory phase CT image using the expiratory phase CT image as a reference, and the iVAS system (ITEM Co., Osaka, Japan) was employed to derive the displacement vector and map it to the expiratory phase CT image [16,17]. DIR is a non-affine process that uses a mathematical model to deform one image to match another. Unlike rigid or affine registration, DIR assigns a deformation vector to each voxel, with the movement loosely dependent on neighboring voxels [18-20]. For the Jacobian metric, the Jacobian determinant V(x, y, z) of the displacement vector u is expressed as follows:

\(v(x, y, z)=\begin{vmatrix} 1+\frac{∂u_{x}(x, y, z)}{∂x} &\frac{∂u_{x}(x, y, z)}{∂y} & \frac{∂u_{x}(x, y, z)}{∂z} \\ \frac{∂u_{y}(x, y, z)}{∂x}& 1+\frac{∂u_{y}(x, y, z)}{∂y} &\frac{∂u_{y}(x, y, z)}{∂z} \\ \frac{∂u_{z}(x, y, z)}{∂x} & \frac{∂u_{z}(x, y, z)}{∂y} & 1+\frac{∂u_{z}(x, y, z)}{∂z} \end{vmatrix}\)

**Figure 1:**
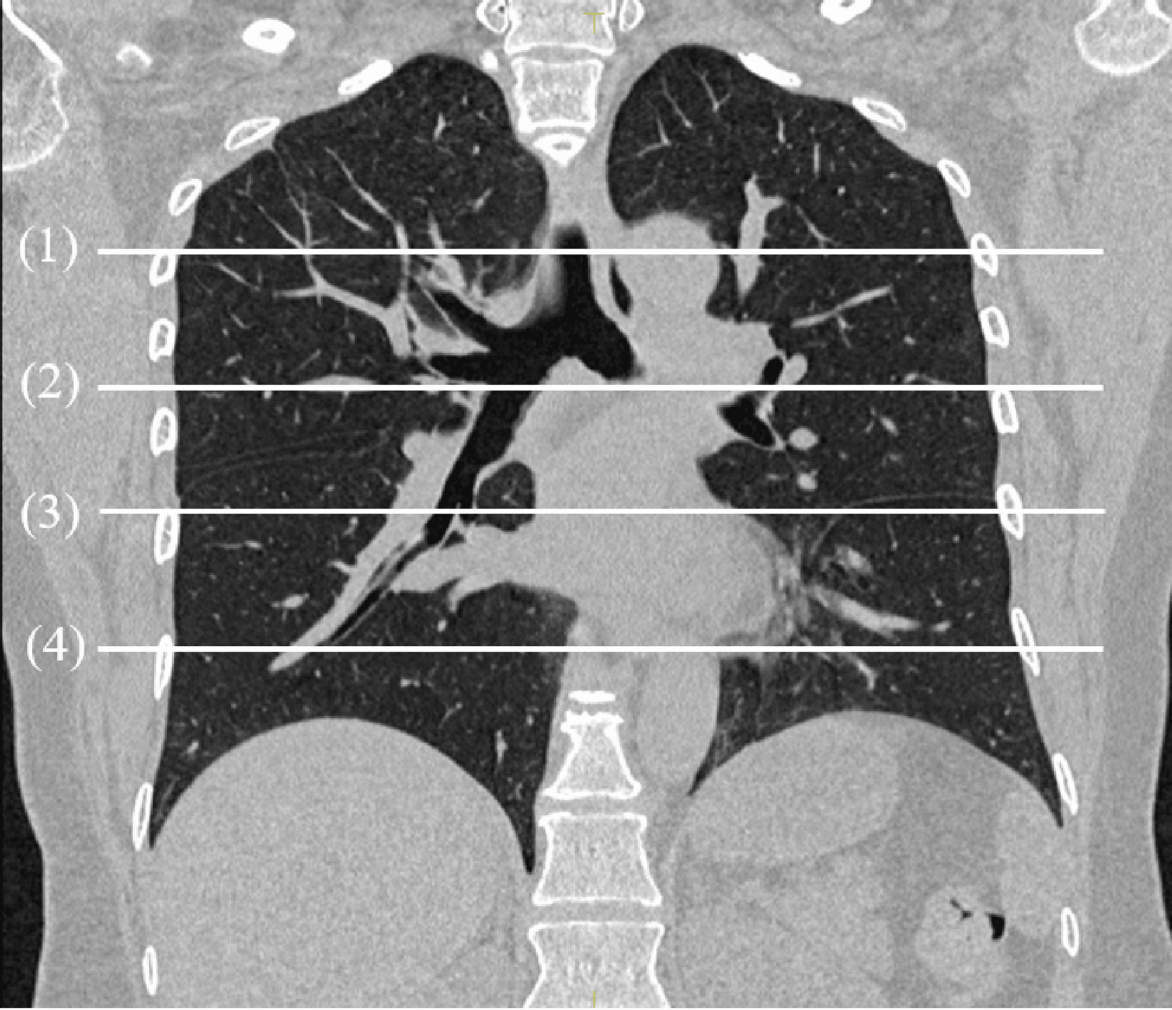
Slice positions for assessing the proportion of highly functional areas in each position (supine, prone, right lateral, and left lateral). (1) Apical: arch level, (2) middle: bifurcation level, (3) basal: heart level, (4) diaphragm.

The Jacobian determinant indicates local volume expansion or contraction at a location (x, y, z) within the image. A determinant value greater than 1 signifies local tissue expansion, while a value less than 1 signifies local tissue contraction. A value of 1 indicates no expansion or contraction [16,17]. Lung ventilation images, calculated using the Jacobian metric based on B-spline registration, were displayed as percentiles. Areas in the 75th percentile or higher were defined as highly functional [11,16,21]. This method provides a clear delineation of functional structures and allows for a continuous distribution of functional weighting for each voxel in the lungs. We compared the proportion of highly functional areas across four lung sections (apical, middle, basal, and diaphragm) (Figure 1) between different positions (prone, right lateral, and left lateral), using the supine position as the reference.

DVF imaging was generated using DIR-based BH-CTVI, which calculated the displacement vector from expiration to inspiration for each lung lobe. All data were manually annotated using the open-source software package vv and 4D Slicer (https://github.com/open-vv/vv.31). The DVF vectors were visualized as green arrows superimposed on fixed images, with the color representing the magnitude of the deformation vector. We compared the DVF images across the four lung sections (apical, middle, basal, and diaphragm) between different positions (prone, right lateral, and left lateral), using the supine position as the reference.

Statistical analysis

Data were expressed as mean ± standard deviation. Statistical analysis was performed using EZR version 1.50 (Saitama Medical Center, Jichi Medical University, Saitama, Japan; https://www.jichi.ac.jp/saitama-sct/SaitamaHP.files/statmedEN.html). Pairwise comparisons were conducted using the Wilcoxon signed-rank test. The statistical significance threshold was adjusted to 0.016 using Bonferroni’s correction.

## Results

Lung volume in different body positions during inspiration and expiration

Table 2 presents the mean volumes and standard deviations for the total, left, and right lungs during expiration and inspiration under tidal breathing in the supine, prone, right lateral, and left lateral positions. Overall, the total lung volumes for both expiration and inspiration were smallest in the supine position and largest in the lateral decubitus positions. Specifically, the right lung had the largest volumes during both the inspiratory and expiratory phases in the left lateral decubitus position, while the left lung had the largest volumes in the right lateral decubitus position.

**Table 2:** Lung volume in each body position for inspiration and expiration (mean ± SD).

		Supine (mL)	Prone (mL)	Right lateral decubitus (mL)	Left lateral decubitus (mL)
Inspiration	Total lungs	3996.0 ± 958.9	4138.2 ± 886.0	4276.6 ± 984.3	4344.3 ± 978.2
	Right lung	2143.5 ± 509.3	2205.0 ± 499.9	1922.5 ± 388.9	2652.2 ± 636.0
	Left lung	1852.5 ± 453.7	1933.1 ± 396.5	2354.0 ± 610.4	1692.0 ± 353.2
Expiration	Total lungs	3069.9 ± 793.0	3313.5 ± 849.6	3628.5 ± 922.4	3683.4 ± 911.7
	Right lung	1665.0 ± 405.2	1796.0 ± 446.5	1520.2 ± 343.7	2363.0 ± 615.3
	Left lung	1404.9 ± 391.6	1517.5 ± 405.7	2108.3 ± 593.9	1320.3 ± 306.6

Comparison of lung ventilation images across positions

**Figure 2:**
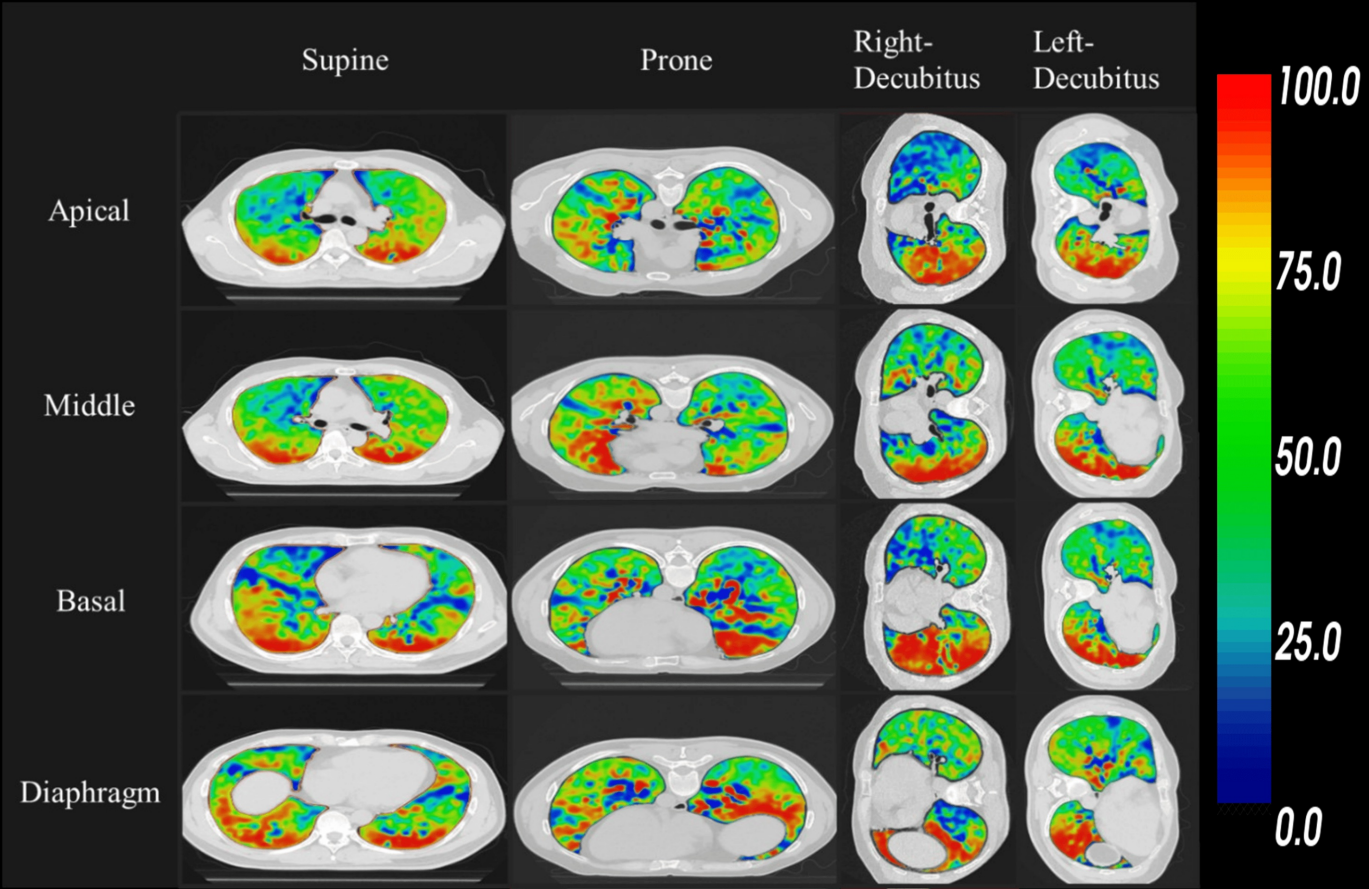
Comparison of lung ventilation images in each position by deformable image registration-based breath hold CT ventilation imaging (BH-CTVI) in the expiration and inspiration phase.

A typical example of lung ventilation images for each body position obtained using DIR-based BH-CTVI at various lung levels (apical, middle, basal, and diaphragm) is shown in Figure 2. In the supine position, highly functional areas were predominantly located in the dorsal region, whereas in the prone position, these areas shifted to the ventral region. In the right lateral decubitus position, highly functional areas were concentrated in the right lung, while in the left lateral decubitus position, they were more prominent in the left lung.

Proportion of highly functional areas in different positions

For each section (apical, middle, basal, and diaphragm) of the left and right lungs, the Jacobian values were converted into percentiles. Table 3 shows the mean and standard deviation of partial lung volumes with values above the 75th percentile, corresponding to the red areas in Figure 2. In all sections, the right lung exhibited the highest proportion of highly functional areas in the right lateral decubitus position, while the left lung showed the highest proportion in the left lateral decubitus position. In the apical section, a significant difference was observed between the right lung volumes in the supine and prone positions. However, no significant differences were observed between the supine and prone positions in other sections for either lung. The proportion of highly functional areas increased in sections closer to the diaphragm compared to those closer to the apex of the lung.

**Table 3:** Ratio of highly functional areas in each position by deformable image registration-based breath hold CT ventilation imaging (BH-CTVI) in the expiration and inspiration phase (mean ± SD). n.s.: not significant.

Cranio-caudal division	Location of ROI	Supine (%)	Prone (%)	Right lateral decubitus (%)	Left lateral decubitus (%)	Supine vs. prone	Supine vs. RD	Supine vs. LD
Apical	Right lung	8.5 ±4.9	16.7 ± 4.1	42.6 ± 10.4	2.7 ± 3.2	p < 0.01	p < 0.01	n.s.
	Left lung	16.7 ± 11.4	22.4 ± 5.9	2.1 ± 1.9	39.5 ± 12.5	n.s.	p < 0.01	p < 0.01
Middle	Right lung	18.8 ± 7.9	21.3 ± 3.9	53.99 ± 13.7	5.5 ±4.5	n.s.	p < 0.01	p < 0.01
	Left lung	18.2 ± 9.0	28.8 ± 9.6	3.72 ± 2.5	54.9 ± 12.9	n.s.	p < 0.01	p < 0.01
Basal	Right lung	31.0 ± 6.7	24.1 ± 8.1	58.3 ± 11.3	7.4 ±5.7	n.s.	p < 0.01	p < 0.01
	Left lung	30.2 ± 12.1	30.2 ± 11.1	5.8 ± 6.1	65.8 ± 14.2	n.s.	p< 0.01	p < 0.01
Diaphragm	Right lung	36.5 ± 4.6	29.1 ± 5.6	65.5 ± 9.8	9.5 ±4.9	n.s.	p < 0.01	p < 0.01
	Left lung	39.8 ± 10.8	26.9 ± 11.4	9.3 ± 7.8	70.4 ± 13.8	n.s.	p < 0.01	p < 0.01

Comparison of DVF imaging across positions

**Figure 3:**
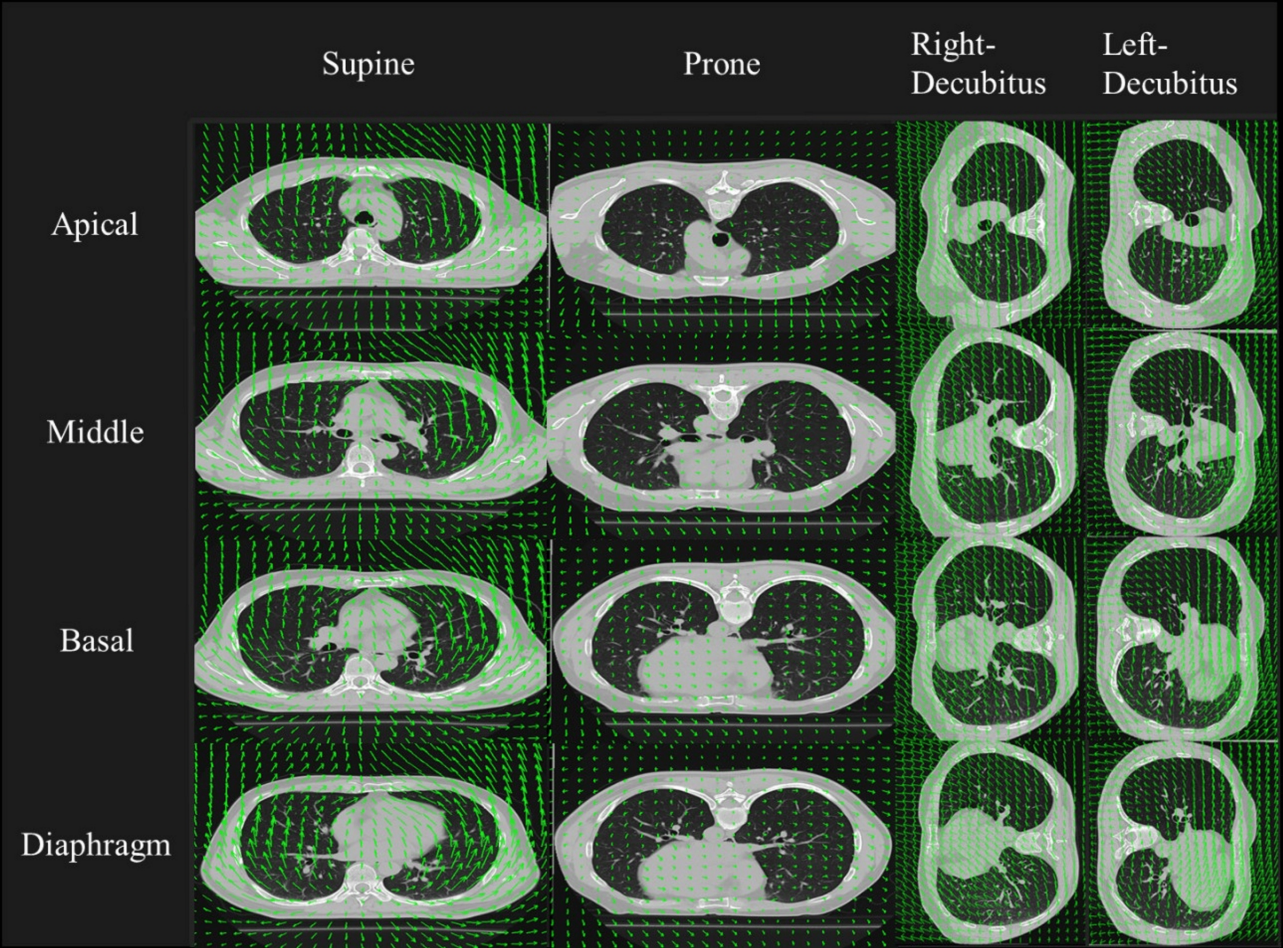
Comparison of deformable vector field (DVF) imaging in each position by deformable image registration (DIR)-based breath hold CT ventilation imaging (BH-CTVI) using expiration and inspiration phase.

Figure 3 illustrates the DVF imaging for each body position, obtained using DIR-based BH-CTVI. In the supine position, the displacement vectors were oriented from the dorsal to the ventral direction. In the prone position, the vectors displayed a nearly parallel orientation to the ground, directed from the head to the feet. In both lateral decubitus positions, the displacement vectors exhibited various orientations.

## Discussion

In this study, we investigated the impact of body position on lung ventilation and DVF imaging using DIR-based BH-CTVI across supine, prone, right lateral decubitus, and left lateral decubitus positions. Our analysis focused on the changes in lung ventilation images and DVF images due to positional transformation. When evaluating pulmonary function using DIR-based BH-CTVI, it is important to evaluate DVF images in addition to ventilation images to comprehensively understand pulmonary dynamics. The results showed that the proportion of highly functional areas was largest in the right lung when in the right lateral decubitus position and in the left lung when in the left lateral decubitus position. These highly functional areas consistently appeared on the side of the body in contact with the ground, correlating with the direction of gravitational force. In contrast, the DVF images revealed that, except for the prone position, the displacement vectors in all other positions were not uniform and varied in orientation. Only in the prone position were the displacement vectors aligned parallel to the ground, suggesting that this position is less influenced by gravity. This finding implies that the prone position may be beneficial for improving lung function in patients with severe ARDS.

The lung volumes were smallest in the supine position and largest in both lateral decubitus positions. Specifically, the left lung volume was greatest in the right lateral decubitus position and smallest in the left lateral decubitus position. These variations are likely due to the gravitational forces that deform the lung and alter the position and shape of the heart, leading to changes in lung volume. As discussed in the Results section, the apical section of the right lung had a smaller ratio of highly functional areas in the supine position. There have been no suggestions in the literature regarding pressure changes influencing these areas. The lung apex is naturally a region with limited movement, and since the right lung has three lobes compared to two in the left lung, the effect of pressure from the abdomen (diaphragm) is thought to have a smaller impact.

In the lung ventilation images, regions with Jacobian values in the 75th percentile or higher, indicating highly functional areas, were consistently located on the side of the body in contact with the ground. This distribution is likely influenced by gravitational forces. Kipritidisa et al. reported similar findings in xenon-CT ventilation images of sheep (in the supine position), where regions with ventilation values in the 90th percentile or higher were dorsally located, driven by gravitational effects [21]. Furthermore, Gattinoni et al. reported that transitioning from a supine to prone position reduces the gravity-induced increase in dorsal lung tissue density, increasing the homogeneity of lung tissue density and thereby improving lung ventilation [22]. This suggests that minimizing the impact of gravity on lung motion is crucial for preserving lung function, especially in patients with severe ARDS. Although our comparison of lung ventilation images using DIR-based BH-CTVI did not show a distinct advantage for the prone position, the maintenance of lung function in the left lung field in the right lateral decubitus position and in the right lung field in the left lateral decubitus position suggests that lateral decubitus positioning may be appropriate when lung disease is localized to one side. In such cases, placing the affected lung in the position least burdened by gravity may optimize lung function. However, gas exchange is determined by the complex interplay of alveolar ventilation, diffusion, and perfusion [23]. Therefore, determining the optimal positioning to preserve lung function in patients with severe ARDS remains challenging.

The DVF images obtained by DIR-based BH-CTVI represent the displacement of lung lobes from expiration to inspiration in vector form. When comparing the DVF images across positions, in the supine position, the displacement vectors are directed opposite to the force of gravity. In contrast, in both lateral decubitus positions, the displacement vectors are oriented in various directions. Only in the prone position were the vectors consistently oriented parallel to the ground, from head to feet (Figure 3). In the prone position, the abdomen rests in contact with the ground and is subjected to constant pressure, while the back is supported by the spine and enclosed by the ribs and other rigid structures. Consequently, lung lobe movement during ventilation is constrained in a single direction and, therefore, less affected by gravitational forces. In the supine and lateral decubitus positions, the lack of rigid structures on the ventral side and differences in diaphragmatic or thoracic breathing likely contribute to the irregular movement of the lung lobes. Therefore, the DVF image comparison indicates that the prone position, being the least affected by gravity, is advantageous for preserving lung function in patients with severe ARDS.

This study has several limitations. First, we did not investigate the correlation between DIR-based BH-CTVI and lung function. Second, we did not examine DIR-based BH-CTVI using different algorithms, and we focused solely on healthy participants. Kipritidisa et al. reported that both the choice of CTVI algorithm and the presence and type of lung disease, as well as the imaging modality used for reference ventilation, can impact the correlation between DIR-based BH-CTVI and lung function [21]. However, the prone position is typically only beneficial in severe ARDS cases [23]. While this study focused on positional changes and their effects on lung ventilation and DVF images in healthy subjects, future research should target patients with reduced lung function, such as those with chronic obstructive pulmonary disease or idiopathic pulmonary fibrosis (IPF).

## Conclusions

This study demonstrated that the prone position facilitates lung ventilation with the least gravitational impact compared to the supine, right lateral decubitus, and left lateral decubitus positions, based on DIR-based BH-CTVI data. When assessing lung function using DIR-based BH-CTVI, it is crucial to evaluate DVF images in addition to ventilation images to gain a comprehensive understanding of lung dynamics.
